# Supplementary material for: The genome sequence of the Square-spot Deerfly, Chrysops viduatus (Fabricius, 1794) (Diptera: Tabanidae)
Source: Wellcome Open Res. 2025 Nov 21;10:650. [Version 1] doi: 10.12688/wellcomeopenres.25193.1 (PMC12824486; doi:10.12688/wellcomeopenres.25193.1)
Supplement: Peer review history

# Reviewer report: Reviewer response for version 1

**DOI:** 10.21956/wellcomeopenres.27769.r142531

**Published:** 2026-01-21

**Authors:** Arun Arumugaperumal

**Affiliations:** 1 Rajalakshmi Engineering College, Chennai, Tamil Nadu, India

The authors have sequenced the genome of Square-spot Deerfly *Chrysops viduatus*. The assembly reported here is of size 312.35 Mb. They have used PacBio long-read sequencing and Hi-C sequence information to obtain a high-quality genome assembly. The genome has 5 chromosomes. The sex chromosomes have not been identified because of technical difficulty. The mitochondrial genome has also been assembled of size 16.08 kb. The quality of the assembly is evident from the high N50 values. BUSCO analysis has shown that the assembly is 93.2% complete with reference to the diptera_odb10 dataset. The data note can be indexed.

Are sufficient details of methods and materials provided to allow replication by others?

Yes

Is the rationale for creating the dataset(s) clearly described?

Yes

Are the datasets clearly presented in a useable and accessible format?

Yes

Are the protocols appropriate and is the work technically sound?

Yes

Reviewer Expertise:

Bioinformatics; Genomics

I confirm that I have read this submission and believe that I have an appropriate level of expertise to confirm that it is of an acceptable scientific standard.

# Reviewer report: Reviewer response for version 1

**DOI:** 10.21956/wellcomeopenres.27769.r142530

**Published:** 2026-01-02

**Authors:** Chao Du

**Affiliations:** 1 Baotou Teachers College, Sichuan Academy of Agricultural Sciences, Baotou, China

This article presents a well-structured and comprehensive account of the genome sequencing of *Chrysops viduatus* (Square-spot Deerfly). It provides valuable reference material for the Darwin Tree of Life project. The background section offers detailed insights into the species' classification, distribution, life habits, and developmental characteristics, which are crucial for understanding the research context. The methods described are appropriate and clearly outlined. Furthermore, the openness of the data contributes to scientific communication and verification. In conclusion, this is a high-quality DATA NOTE that provides genomic data for *Chrysops viduatus*, significantly contributing to research on this species.

Are sufficient details of methods and materials provided to allow replication by others?

Yes

Is the rationale for creating the dataset(s) clearly described?

Yes

Are the datasets clearly presented in a useable and accessible format?

Yes

Are the protocols appropriate and is the work technically sound?

Yes

Reviewer Expertise:

Genomics

I confirm that I have read this submission and believe that I have an appropriate level of expertise to confirm that it is of an acceptable scientific standard.

# Reviewer report: Reviewer response for version 1

**DOI:** 10.21956/wellcomeopenres.27769.r141614

**Published:** 2025-12-17

**Authors:** Stuart J.E. Baird

**Affiliations:** 1 Academy of Sciences of the Czech Republic, Národní, Staré Město, Czech Republic

This genome report differs from previous I have reviewed in three ways: first, different individuals were used for the Hi-C and PacBio sequencing; second, there are two regions where the order and orientation of scaffolds is uncertain; third the percentage of the assembly scaffolded to chromosomes is about 10% lower than appears usual for DToL. This despite 170x PacBio coverage, and 3.5x more Gb of Hi-C than PacBio.

 The genome is of course worth indexing. What is lacking in the report is some comparative commentary which would allow readers of these reports to understand the sources of variance in final outcome. eg is the repeat content of 27.47% a potential explanation? Are the unordered/unoriented regions particularly rich in repeats?

Are sufficient details of methods and materials provided to allow replication by others?

Yes

Is the rationale for creating the dataset(s) clearly described?

Yes

Are the datasets clearly presented in a useable and accessible format?

Yes

Are the protocols appropriate and is the work technically sound?

Yes

Reviewer Expertise:

Admixture genomics, spatial genetics

I confirm that I have read this submission and believe that I have an appropriate level of expertise to confirm that it is of an acceptable scientific standard, however I have significant reservations, as outlined above.
